# Supplementary material for: Interventions promoting recovery from depression for patients transitioning from outpatient mental health services to primary care: A scoping review
Source: PLoS One. 2024 May 6;19(5):e0302229. doi: 10.1371/journal.pone.0302229 (PMC11073719; doi:10.1371/journal.pone.0302229)
Supplement: S1 Appendix — (DOCX) [file pone.0302229.s001.docx]

# **S1 Appendix**

## **List of abbreviations/concepts**

| Abbreviation / concept | Definition |
| --- | --- |
| MDD | Major Depressive Disorder |
| Outpatient mental health services | Outpatient hospital-based mental health services |
| DSM-IV | The Diagnostic and Statistical Manual of Mental Disorders, Fifth Edition |
| ICD-10 | International Classification of Diseases and Related Health Problems 10^th^ Revision |
| GP | General practitioner |
| RCT | Randomized Controlled Trial |
| CRT | Circadian Reinforcement Therapy |
| Co-design | A participatory approach to design interventions in collaboration with stakeholders. |
| JBI | Joanna Briggs Institute |
| PRISMA-ScR | Preferred Reporting Items for Systematic Reviews and Meta-Analysis - extension for Scoping Reviews checklist |
| Stakeholder | Any individual or group who is responsible for or affected by health- and healthcare-related decisions that can be informed by research evidence [1]*.* In this study stakeholders are patients, general practitioners, psychiatrists, nurses, job-consultants, medical social workers, and researchers. |
| Patients transitioning | When patients move between care settings. In this study, we focus on patients’ transition from outpatient mental health services to primary care |
| RQ | Research Question |
| MeSh | Medical Subject Headings |
| TIDieR | Template for Intervention Description and Replication |
| BDI-II | Beck Depression Inventory |
| HAM-D | Hamilton Depression Rating Scale |
| MADRS | Montgomery & Åsberg Depression Rating Scale |
| PSR | Psychiatric Rating Scale |
| PHQ | Patient Health Questionnaire |
| RAS | Recovery Assessment Scale |
| SCL | Symptom Checklist Depression Scale |
| DBS | Deep Brain Stimulation |
| CBT | Cognitive Behavioural Therapy |
| LiCBT | Low-intensity Cognitive Behavioral Therapy |
| C-CT | Continuation-phase Cognitive Therapy |
| CBT-TT | Cognitive Behavioural Therapy Telephone Treatment |
| FLX | Fluoxetine |
| PBO | Placebo |
| MCT | Metacognitive Therapy |
| IPT | Interpersonal Psychotherapy |
| RFCBT | Rumination Focused Cognitive Behavioural Therapy |
