# Supplementary material for: Interventions promoting recovery from depression for patients transitioning from outpatient mental health services to primary care: A scoping review
Source: PLoS One. 2024 May 6;19(5):e0302229. doi: 10.1371/journal.pone.0302229 (PMC11073719; doi:10.1371/journal.pone.0302229)
Supplement: S3 Appendix — (DOCX) [file pone.0302229.s003.docx]

# **S3 Appendix**

## **Eligibility criteria**

| PICOS | Inclusion | Exclusion |
| --- | --- | --- |
| Population | Adults (18 years of age or older)  with the primary diagnosis of  MDD (as diagnosed using any  recognized diagnostic criteria,  e.g., DSM-IV or ICD-10, S1  Appendix). We include studies of  patients with co-occurring.  Disorders if they have a primary  diagnosis of MDD. | Studies with exclusively elderly people (65 years of age or older), psychotic depression, depression as part of bipolar disorder, or people suffering exclusively from postpartum depression. Co-occurring alcohol or drug abuse, personality, phobia, or anxiety disorders are not exclusion criteria. |
| Intervention | We include studies investigating any type of intervention e.g., simple, multicomponent, or complex interventions that aim to promote recovery from depression for patients transitioning from outpatient mental health services to primary care. This definition includes interventions that are both pharmacological and non-pharmacological, which can be delivered via the Internet, a website, a mobile-setting, in-person, or a mix thereof. |  |
| Comparator | At this stage, any comparator will be included. In comparator studies, the control group can both receive treatment as usual, a placebo, an active ingrediency or alternative interventions. | *The criteria for comparator do not apply to qualitative studies.* |
| Outcome | Improvement in recovery from MDD. | *The criteria for outcome do not apply to qualitative studies.* |
| Setting | Patients must be in the transitioning setting from outpatient mental health services to primary care. This includes studies in which patients are nearing the end of their outpatient treatment in a mental healthcare setting, or patients who are being treated in primary care after discharge - we will only include studies concerning patients who are being treated in primary care if patients previously have been treated in an outpatient mental health service.  We will include studies in which patients either have been or have not been hospitalized in an inpatient mental health service before their treatment course in an outpatient mental health service. | Patients who have not previously been treated in an outpatient mental health service, or patients who are recruited from an inpatient mental health service. |
