# Supplementary material for: Interventions promoting recovery from depression for patients transitioning from outpatient mental health services to primary care: A scoping review
Source: PLoS One. 2024 May 6;19(5):e0302229. doi: 10.1371/journal.pone.0302229 (PMC11073719; doi:10.1371/journal.pone.0302229)
Supplement: S4 Appendix — (DOCX) [file pone.0302229.s004.docx]

# **S4 Appendix**

## **Search Strategy, PubMed**

("Depressive Disorder"[MeSH Terms] OR "depression"[MeSH Terms]) AND ("Mental Health Services"[MeSH Terms:noexp] OR "Health Care Sector"[MeSH Terms] OR "Organization and Administration"[MeSH Terms] OR "Patient Care Management"[MeSH Terms] OR "Community Health Services"[MeSH Terms] OR "Continuity of Patient Care"[MeSH Terms] OR "General Practice"[MeSH Terms] OR ("mental health service*"[Text Word] OR "primary care"[Text Word] OR "primary health care"[Text Word] OR "secondary care"[Text Word] OR "secondary health care"[Text Word] OR "General Practice"[Text Word] OR "patient discharge"[Text Word] OR "patient transfer*"[Text Word] OR "transitional care"[Text Word] OR "after care"[Text Word] OR "patient care continuity"[Text Word] OR "transition*"[Text Word] OR "health care"[Text Word] OR "Organization"[Text Word])) AND ("Behavioral Disciplines and Activities"[MeSH Terms] OR "Mental Processes"[MeSH Terms] OR "Transtheoretical Model"[MeSH Terms] OR "Therapeutics"[MeSH Terms] OR ("intervention*"[Text Word] OR "method*"[Text Word] OR "model*"[Text Word] OR "procedure*"[Text Word] OR "process*"[Text Word] OR "treatment*"[Text Word] OR "therapy*"[Text Word])) AND ("Mental Health Recovery"[MeSH Terms] OR "Recovery of Function"[MeSH Terms] OR "Return to Work"[MeSH Terms] OR "Return to School"[MeSH Terms] OR "recover*"[Text Word]
