# Supplementary material for: Interventions promoting recovery from depression for patients transitioning from outpatient mental health services to primary care: A scoping review
Source: PLoS One. 2024 May 6;19(5):e0302229. doi: 10.1371/journal.pone.0302229 (PMC11073719; doi:10.1371/journal.pone.0302229)
Supplement: S5 Appendix — (DOCX) [file pone.0302229.s005.docx]

| S5 Appendix Sample Demographics | | | | | | | |
| --- | --- | --- | --- | --- | --- | --- | --- |
| Author and year | Number of participants randomized / included in analysis *(n*=) | Number of participants completed (*n*=) | Mean age in years | Range age in years | Gender – female (%) | Marital status (%) (married/partnered) | Other diagnosis – one or more (%) |
| Callesen et al. (2020) [1] | 174 (R) | 115 | 35 years (B) | 18 years or older | 69% (B) | 36.1% (B) | 30.3% (B) |
| Craigie and Nathan (2009) [2] | 356 (R) | 234 | 36.4 years (W) | Not reported | 65% (W) | 18% (W) | 15.7% (W) |
| Ekeblad et al. (2016) [3] | 96 (R) | 63 | 34.2 years (W) | Not reported | 68.8% (W) | 37.5% (W) | 49.5% (W)  *personality disorder* |
| Tønning et al. (2021) [4] | 120 (R) | 100 | 44 years (B) | Over 18 | 52.5% (B) | 49.2% (B) | 42.5% (B)  *psychiatric comorbidity* |
| Thimm and Antonsen (2014) [5] | 143 (W) | 118 | 41.6 years (W) | 20-69 (W) | 71% (W) | 56% (W) | 31.8% (W)  *patients with ≥2 diagnosis* |
| Ezquiaga et al. (1998) [6] | 90 (W) | 87 | 46.3 years (C) | 18-65 (W) | 77% (C) | Not reported | 28.7% (W)  *personality disorder* |
| Ludman et al. (2016) [7] | 320 (R) | 274 | 49.8 years (B) | 18 years or older | 67.9% (B) | 36.8% (B) | 57% (B)  *borderline personality disorder* |
| Tutty et al. (2010) [8] | 30 (W) | 26 | 33.1 years (W) | 18-65 | 67% (W) | 34% (W) | Not reported |
| Vittengl et al. (2010) [9] | 84 (R) | 55 | 42.7 years (W) | Not reported | 72.6% (W) | Not reported | 17.9% (W)  *social phobia* |
| Jarrett et al. (2013) [10] | 241 (R) | 124 | 42.7 years (W) | Not reported | 67.2% (W) | 42.7% (W) | Not reported |
| Vittengl et al. (2016) [11] | 241 (R) | 124 | 42.7 years (W) | Not reported | 67.2% (W) | 42.7% (W) | Not reported |
| Skärsäter et al. (2005) [12] | 32 (B) | 24 | 42.1 years (B) | 19-60 (B) | 79.2% (C) | 44% (B) | Not reported |
| Steig et al. 2023 [13] | 171 (R) | 101 | 34 years (R) | Not reported | 64.9 (R) | Not reported | 14.6% (R)  *social anxiety disorder* |
| Lawn et al. (2019) [14] | 680 (W) | 427 | 53.8 years (B) | Not reported | 72.8% (B) | 52.5% (B) | 19% (B)  *anxiety disorder* |
| Woolley et al. (2020) [15] | 5 | 5 | 48.4 years | 38-62 years | 100% | 20% | 40%  *generalized anxiety disorder* |
| Peden (1996) [16] | 7 | 7 | 40 years | 30-54 years | 100% | 42.9% | Not reported |
| Skärsäter et al. (2003) [17] | 13 | 13 | 42 years | 28-63 years | 100% | 61.5% | Not reported |
| Bouchal et al. (2023) [18] | 18 | 18 | 47.3 years | Not reported | 44.4 | Not reported | Not reported |

**Completed sample (C), Whole sample (W), Baseline sample (B), Randomized sample (R)**

# **References**

1. Callesen, P., et al., *Metacognitive therapy for bipolar II disorder: A single case series study.* Neurology Psychiatry and Brain Research, 2020. **38**: p. 107-113.

2. Craigie, M.A. and P. Nathan, *A nonrandomized effectiveness comparison of broad-spectrum group CBT to individual cbt for depressed outpatients in a community mental health setting.* Behavior Therapy, 2009. **40**(3): p. 302-314.

3. Ekeblad, A., et al., *Randomized Trial of Interpersonal Psychotherapy and Cognitive Behavioral Therapy for Major Depressive Disorder in a Community-Based Psychiatric Outpatient Clinic.* Depress Anxiety, 2016. **33**(12): p. 1090-1098.

4. Tønning, M.L., et al., *The effect of smartphone-based monitoring and treatment on the rate and duration of psychiatric readmission in patients with unipolar depressive disorder: The RADMIS randomized controlled trial.* J Affect Disord, 2021. **282**: p. 354-363.

5. Thimm, J.C. and L. Antonsen, *Effectiveness of cognitive behavioral group therapy for depression in routine practice.* BMC Psychiatry, 2014. **14**: p. 292.

6. Ezquiga, E., et al., *Factors associated with outcome in major depression: A 6-month prospective study.* Social Psychiatry and Psychiatric Epidemiology: The International Journal for Research in Social and Genetic Epidemiology and Mental Health Services, 1998. **33**(11): p. 552-557.

7. Ludman, E.J., et al., *Organized self-management support services for chronic depressive symptoms: A randomized controlled trial.* Psychiatric Services, 2016. **67**(1): p. 29-36.

8. Tutty, S., et al., *Evaluating the effectiveness of cognitive-behavioral teletherapy in depressed adults.* Behav Ther, 2010. **41**(2): p. 229-36.

9. Vittengl, J.R., L.A. Clark, and R.B. Jarrett, *Moderators of continuation phase cognitive therapy's effects on relapse, recurrence, remission, and recovery from depression.* Behav Res Ther, 2010. **48**(6): p. 449-58.

10. Jarrett, R.B., et al., *Preventing depressive relapse and recurrence in higher-risk cognitive therapy responders: a randomized trial of continuation phase cognitive therapy, fluoxetine, or matched pill placebo.* JAMA Psychiatry, 2013. **70**(11): p. 1152-60.

11. Vittengl, J.R., et al., *Longitudinal social-interpersonal functioning among higher-risk responders to acute-phase cognitive therapy for recurrent major depressive disorder.* J Affect Disord, 2016. **199**: p. 148-56.

12. Skärsäter, I., et al., *Sense of coherence and social support in relation to recovery in first-episode patients with major depression: A one-year prospective study.* International Journal of Mental Health Nursing, 2005. **14**(4): p. 258-264.

13. á Steig, D.H., et al., *Patient-reported outcome measures in depression.* Nordic Journal of Psychiatry, 2023. **77**(2): p. 212-219.

14. Lawn, S., et al., *Outcomes of telephone-delivered low-intensity cognitive behaviour therapy (LiCBT) to community dwelling Australians with a recent hospital admission due to depression or anxiety: Mindstep™.* BMC Psychiatry, 2019. **19**.

15. Woolley, H., et al., *"I'm not alone": Women's experiences of recovery oriented occupational therapy groups following depression.* Can J Occup Ther, 2020. **87**(1): p. 73-82.

16. Peden, A.R., *Recovering from depression: a one-year follow-up.* J Psychiatr Ment Health Nurs, 1996. **3**(5): p. 289-95.

17. Skärsäter, I., et al., *Women's conceptions of coping with major depression in daily life: A qualitative, salutogenic approach.* Issues in Mental Health Nursing, 2003. **24**(4): p. 419-439.

18. Raffin Bouchal, D.S., et al., *Personal recovery associated with deep brain stimulation for treatment-resistant depression: A constructivist grounded theory study.* J Psychiatr Ment Health Nurs, 2023. **30**(5): p. 1005-1018.
