# Supplementary material for: Interventions promoting recovery from depression for patients transitioning from outpatient mental health services to primary care: A scoping review
Source: PLoS One. 2024 May 6;19(5):e0302229. doi: 10.1371/journal.pone.0302229 (PMC11073719; doi:10.1371/journal.pone.0302229)
Supplement: S6 Appendix — (DOCX) [file pone.0302229.s006.docx]

# **S6 Appendix**

## **Rating Scales used to measure recovery outcomes ordered by year of publication (*n* = 14)**

| Author and year | BDI-II | HAM-D | MADRS | PHQ | RAS | SCL | PSR |
| --- | --- | --- | --- | --- | --- | --- | --- |
| Ezquiaga et al. (1998) [1] |  | ● |  |  |  |  |  |
| Skärsäter et al. (2005) [2] |  |  | ● |  |  |  |  |
| Craigie and Nathan (2009) [3] | *●* |  |  |  |  |  |  |
| Tutty et al. (2010) [4] |  |  |  |  |  | ● |  |
| Vittengl et al. (2010) [5] |  |  |  |  |  |  | ● |
| Jarett et al. (2013) [6] |  |  |  |  |  |  | ● |
| Thimm and Antonsen (2014) [7] | ● |  |  |  |  |  |  |
| Ekeblad et al. (2016) [8] | ● |  |  |  |  |  |  |
| Ludman et al. (2016) [9] |  |  |  |  | ● |  |  |
| Vittengl et al. (2016) [10] |  |  |  |  |  |  | ● |
| Lawn et al. (2019) [11] |  |  |  | *●* |  |  |  |
| Callesen et al. (2020) [12] | *●* |  |  |  |  |  |  |
| Tønning et al. (2021) [13] |  |  |  |  | ● |  |  |
| Steig et al. (2023) [14] | *●* |  |  |  |  |  |  |

# **References**

1. Ezquiga, E., et al., *Factors associated with outcome in major depression: A 6-month prospective study.* Social Psychiatry and Psychiatric Epidemiology: The International Journal for Research in Social and Genetic Epidemiology and Mental Health Services, 1998. **33**(11): p. 552-557.

2. Skärsäter, I., et al., *Sense of coherence and social support in relation to recovery in first-episode patients with major depression: A one-year prospective study.* International Journal of Mental Health Nursing, 2005. **14**(4): p. 258-264.

3. Craigie, M.A. and P. Nathan, *A nonrandomized effectiveness comparison of broad-spectrum group CBT to individual cbt for depressed outpatients in a community mental health setting.* Behavior Therapy, 2009. **40**(3): p. 302-314.

4. Tutty, S., et al., *Evaluating the effectiveness of cognitive-behavioral teletherapy in depressed adults.* Behav Ther, 2010. **41**(2): p. 229-36.

5. Vittengl, J.R., L.A. Clark, and R.B. Jarrett, *Moderators of continuation phase cognitive therapy's effects on relapse, recurrence, remission, and recovery from depression.* Behav Res Ther, 2010. **48**(6): p. 449-58.

6. Jarrett, R.B., et al., *Preventing depressive relapse and recurrence in higher-risk cognitive therapy responders: a randomized trial of continuation phase cognitive therapy, fluoxetine, or matched pill placebo.* JAMA Psychiatry, 2013. **70**(11): p. 1152-60.

7. Thimm, J.C. and L. Antonsen, *Effectiveness of cognitive behavioral group therapy for depression in routine practice.* BMC Psychiatry, 2014. **14**: p. 292.

8. Ekeblad, A., et al., *Randomized Trial of Interpersonal Psychotherapy and Cognitive Behavioral Therapy for Major Depressive Disorder in a Community-Based Psychiatric Outpatient Clinic.* Depress Anxiety, 2016. **33**(12): p. 1090-1098.

9. Ludman, E.J., et al., *Organized self-management support services for chronic depressive symptoms: A randomized controlled trial.* Psychiatric Services, 2016. **67**(1): p. 29-36.

10. Vittengl, J.R., et al., *Longitudinal social-interpersonal functioning among higher-risk responders to acute-phase cognitive therapy for recurrent major depressive disorder.* J Affect Disord, 2016. **199**: p. 148-56.

11. Lawn, S., et al., *Outcomes of telephone-delivered low-intensity cognitive behaviour therapy (LiCBT) to community dwelling Australians with a recent hospital admission due to depression or anxiety: Mindstep™.* BMC Psychiatry, 2019. **19**.

12. Callesen, P., et al., *Metacognitive Therapy versus Cognitive Behaviour Therapy in Adults with Major Depression: A Parallel Single-Blind Randomised Trial.* Sci Rep, 2020. **10**(1): p. 7878.

13. Tønning, M.L., et al., *The effect of smartphone-based monitoring and treatment on the rate and duration of psychiatric readmission in patients with unipolar depressive disorder: The RADMIS randomized controlled trial.* J Affect Disord, 2021. **282**: p. 354-363.

14. á Steig, D.H., et al., *Patient-reported outcome measures in depression.* Nordic Journal of Psychiatry, 2023. **77**(2): p. 212-219.
