# Supplementary material for: Interventions promoting recovery from depression for patients transitioning from outpatient mental health services to primary care: A scoping review
Source: PLoS One. 2024 May 6;19(5):e0302229. doi: 10.1371/journal.pone.0302229 (PMC11073719; doi:10.1371/journal.pone.0302229)
Supplement: S8 Appendix — (DOCX) [file pone.0302229.s008.docx]

# **S8** **Appendix**

## **Content of interventions (*n*=16)**

| Author and year | Name of intervention | Materials | Procedures* | Duration | Professional | Delivery method | Tailoring |
| --- | --- | --- | --- | --- | --- | --- | --- |
| Callesen et al. (2020) [1] | Metacognitive therapy (MCT) and cognitive behavioral therapy (CBT) | MCT protocol: Standard published treatment manual by Wells [2]  CBT protocols: Manuals by Beck et al. [3] supplemented by Fennel’s protocol [4], and NICE recommendations. | MCT: Patients learned about MCT strategies and received a booklet with homework assignments.  CBT: Patients learned about CBT strategies and received a booklet with homework assignments. | Up to 24 sessions of MCT or CBT. Each session lasted 60 minutes. | The same two clinical psychologists and CBT therapists with a minimum of 10 years’ experience delivered treatment. | Face-to-face | Decisions to terminate treatment were based on patients achieving a score of ≤8 on the BDI II on two consecutive occasions and/ agreeing on termination. |
| Craigie and Nathan (2009) [5] | Group cognitive behavioral therapy (CBT) or individual CBT. | Group CBT protocol: A structured program for depression based on Beck et al’s [3] manual for depression and Barlow and Cracke’s [6] manual for anxiety.  Individual CBT protocol: Described in Nathan et al. [7]  Also employed elements based on Beck et al’s [3] manual for depression. | CBT: Patients learned about CBT techniques and received homework tasks, e.g., graded exposure, diaries, psychoeducation, and calming techniques. Agenda and activities for each session included standard handouts, and worksheet materials. | Group CBT: 10 weekly 2-hour group sessions, with a 1-month follow-up session.  Individual CBT: Not reported. | Therapists had at least masters-level clinical psychology training in CBT and clinical assessment and were qualified clinical psychologists, clinical psychologist registrars, or clinical psychology master-level students. | Group or individual | Clinicians implemented individual CBT in a more flexible manner based on a cognitive-behavioral care formulation for each patient. Patients who were unwilling to take part in group CBT were offered individual treatment. |
| Ekeblad et al. (2016) [8] | Cognitive behavioral therapy (CBT) or interpersonal psychotherapy (IPT) | IPT protocol: The standard manual by Weissman et al. [9].  CBT protocols: Beck et al. [3] and Martell et al. [10]. | Not reported. | The duration of both treatments was 14 sessions. | Nine IPT therapists: Six psychiatric nurses, one nurse assistant, one occupational therapist, and one social worker.  25 CBT therapists: Psychologists, psychiatric nurses, social workers, nurse assistant, occupational therapist, and physicians. | Not reported. | Some CBT therapists included components of mindfulness in their CBT treatment. |
| Tønning et al. (2021) [11] | A smartphone-based monitoring and treatment system | Smartphone application (app), Monsenso. | Intervention group: Patients filled in subjective data into the smartphone-app that also automatically collected objective data. Patients discussed the data with the nurse. Patients received both text and cartoons for psychoeducation. A study nurse checked the data three times a week and reacted according to the data presented, providing a double feedback loop between the study nurse and the patient.  Control group: Patients received standard treatment and the smartphone-app automatically collected objective data. | Six months following discharge from hospitalization. Patients were encouraged to fill in the daily self-rating, which would take 2-5 minutes. | A study nurse, an experienced psychiatric nurse. | Smartphone app with clinical feedback provided by a study nurse. | Based on the patient’s needs, skills, and clinical status, the app was used on an individual level by the study nurse on how to best help the patient. |
| Thimm and Antonsen (2014) [12] | Cognitive behavioral therapy (CBT) | CBT protocol: Based on the manual by Hagen and Gråwe [13] available in Norway. | Patient learned about cognitive restructuring, psychoeducation, and focus on interpersonal relationships, social network, resources, and pleasurable activities. Approx. 3 months after the last treatment, patients received a group session on treatment evaluation and relapse prevention. | 5–7 patients were included in the CBT-groups. The treatment consisted of 12 weekly sessions, later extended to 15 sessions. Each session lasted 120 minutes. | CBT-groups were led by a therapist and a co-therapist. The therapists were mainly psychiatric nurses, but also other mental professionals (e.g., psychologists). | Group. | As no single manual was used, there was some variation in the treatment. However, the core elements of CBT for depression were central to all treatments. |
| Ezquiaga et al. (1998) [14] | Pharmacological | Antidepressant medication. | Medication had to be used in effective doses. If a favourable response has not been obtained after two months, the medication would be changed to another antidepressant. | Not reported. | Not reported. | Not reported. | Not reported. |
| Ludman et al. (2016) [15] | Self-management support service | Intervention group: Attended an engagement session guided by Grote and colleagues [16] and Zuckoff and colleagues [17] followed by a manualized self-management group program: Adapted from the cognitive behavioral therapy program used in the pilot study (Ludman et al. [18] and supplemented by recovery-oriented content [19, 20].  The recovery model focused on delivery of peer support [21]. | Intervention group: Patients identified, planned, and troubleshot actions to meet personal self-management goals, and received a script that employed evocative and motivational strategies to increase engagement. The intervention focused on the “recovery model”, i.e., receiving peer-support to improve patients’ enjoyment of life by promoting a sense of well-being, hope, and optimism.  Control group: Treatment as usual. | 18-month intervention. Participants met weekly for 10 weeks, twice a month for two months, and then once a month for maintenance of progress. | Four care managers (a social worker, a marriage, a family therapist, a health educator, and a psychologist) delivered telephone contacts, and three peer specialists acted as both a co-facilitator and a participant role model for the groups.  All care managers had experience working with patients with depression, and all peer specialists had personal experience of depression. | Telephone in-person contacts and a structured group program. | Care managers contacted patients at specified intervals no longer than a month apart during the first 12 months, after which the intervals between contacts varied according to symptoms, treatment adherence, and patient engagement. |
| Tutty et al. (2010) [22] | Cognitive behavioural therapy-telephone treatment (CBT-TT) | CBT-TT protocol: Based on earlier telephone CBT trials showing efficacy [23] and durability [24]. Sessions were influenced by motivational interviewing [25, 26]. | Sessions focused on psychoeducation and cognitive-behavioural experimentation in a context influenced by motivational interviewing. | The protocol consisted of eight, 30-minute sessions delivered weekly, followed by two, 30-minute booster sessions delivered at 30-days intervals thereafter. | One study therapist, a doctoral student in clinical psychology. | Telephone in-person counselling’s. | Not reported, however, clients who received CBT-TT were free to seek any other type of additional care over the course of study treatment. |
| Vittengl et al. (2010) [27] | Continuation-phase cognitive therapy (CT) | Patients consented to an acute-phase protocol by Beck et al. [3]. Responders to acute-phase CT were randomized to continuation-phase CT, described by Jarrett et al. in Whisman et al. (2008);[28], or assessment control. | Acute-phase CT and continuation-phase CT: Patients received CBT content, including testing, eliciting thoughts associated with dysphoria, and generating more realistic thoughts.  Assessment control: Evaluation visits. Evaluators of control patients had not provided acute-phase CT and did not use psychosocial interventions. | 12-14 weeks acute-phase CT: 20 sessions (50-60 minutes each).  8-month continuation-phase CT or assessment control: 10 sessions (60 minutes each).  Follow-up: 10 assessments over 16 months. | Five therapists with a PhD in clinical psychology or psychiatry, completed ≥ 1 year of CT training and demonstrated competence (scores ≥ 40 on the Cognitive Therapy Scale; Young & Beck, 1980).  Continuation-phase CT was provided by each patient’s acute-phase CT therapist. | Individual sessions. | Sessions in continuation-phase CT could last up to 90 minutes if necessary.  Patients who relapsed were asked to complete all sessions and, if not receiving continuation-phase CT, were referred for treatment outside of the study. |
| Jarrett et al. (2013) [29] | Continuation-phase cognitive therapy CT (C-CT), clinical management plus fluoxetine (FLX), or pill placebo (PBO) | Acute Phase CT protocol: Based on the treatment manual by Beck et al. [3]. Continuation Phase CT (C-CT): Protocol is described by Jarrett et al. in Whisman et al. (2008);[28], Clinical Management Plus fluoxetine (FLX) or pill placebo (PBO): Clinical management was provided according to [30]. | C-CT: Patient received CBT content, including symptom-reducing cognitive-behavioural techniques, improve coping with adversity, and decrease the probability of stressful events. 8 Month Clinical Management Plus FLX / PBO: Clinicians focused on supportive contact around signs and symptoms, beneficial and untoward effects of the medication, and information about depression. Fluoxetine was chosen.  24 Month Follow-up Phase: Treatments were discontinued every 4 months for 24 months. | Acute Phase CT: 16-20 sessions over 12 weeks (8 twice-weekly sessions). Patients who had obtained reduction began weekly sessions. All patients received two psychoeducational sessions. C-CT: 10-sessions with 4 bi-weekly then 6 monthly sessions (60 minutes each). Clinical Management + FLX / PBO: Initial 30–45-minute session, and then a 15–30-minute session at the same frequency as C-CT. | With few exceptions, the same therapist conducted the acute and continuation phases of CT.  Cognitive therapists: 15 therapists, mostly PhDs, one was an MD.  Clinical Management + FLX / PBO: Experienced pharmacotherapeutics.  Seven male psychiatrists prescribed double-blinded FLX or PBO. | Acute Phase CT and C-CT: Individual sessions.  Clinical Management + FLX / PBO:  Pharmacies at each site packaged and dispensed active FLX or identical PBO capsules. | Acute Phase CT: Up to 2 additional weeks were permitted. Patients with ≥ 40% reduction in HRSD scores received 8 weekly sessions (16 total sessions), whereas patients with less reduction in HRSD scores received 8 twice-weekly sessions before 4 weekly sessions. 8 Month C-CT: The more symptomatic the patient, the more the therapist structured the session. 8 Month Clinical Management Plus FLX / PBO: The dose could vary between 10 mg/day to 40mg/day. Patients who did not tolerate any dose would be followed for clinical management alone. |
| Vittengl et al. (2016) [31] | Continuation-phase cognitive therapy CT (C-CT), clinical management plus fluoxetine (FLX), or pill placebo (PBO) | Acute Phase CT protocol: Based on the treatment manual by Beck et al. [3]. Continuation Phase CT (C-CT): Protocol described by Jarrett et al. in Whisman et al. (2008);[28], Clinical Management Plus fluoxetine (FLX) or pill placebo (PBO): Clinical management was provided according to Fawceet et al. [30]. | C-CT: Patients received CBT content, including symptom-reducing cognitive-behavioural techniques, improve coping with adversity, and decrease the probability of stressful events.  8 Month Clinical Management Plus FLX / PBO: Clinicians focused on supportive contact around signs and symptoms, beneficial and untoward effects of the medication, and information about depression. Fluoxetine was chosen.  24 Month Follow-up Phase Treatments were discontinued every 4 months for 24 months. | Acute Phase CT: 16-20 sessions over 12 weeks (8 twice-weekly sessions). Low Risk Patients received 8 weekly sessions (16 total sessions), whereas High Risk Patients received 8 twice-weekly sessions before 4 weekly sessions (20 total sessions). C-CT: A 10-sessions with 4 bi-weekly then 6 monthly sessions (60 minutes each). Clinical Management + FLX / PBO: Initial 30–45-minute session, and then a 15–30-minute session at the same frequency as C-CT. | Acute Phase CT and C-CT: Patients’ acute phase and C-CT therapists were the same, with few exceptions. The CT was provided by 15 experienced therapists from two sites.  Clinical Management + FLX / PBO: Experienced pharmacotherapeutics provided FLX and PBO. | Acute Phase CT and C-CT: Individual sessions.  Clinical Management + FLX / PBO: Pharmacies dispensed identical capsules with active fluoxetine or placebo. | Acute Phase CT: Up to 2 additional weeks were permitted. Patients with ≥ 40% reduction in HRSD scores received 8 weekly sessions (16 total sessions), whereas patients with less reduction in HRSD scores received 8 twice-weekly sessions before 4 weekly sessions. 8 Month C-CT: The more symptomatic the patient, the more the therapist structured the session. 8 Month Clinical Management Plus FLX / PBO: The dose could vary between 10 mg/day to 40mg/day. Patients who did not tolerate any dose would be followed for clinical management alone. |
| Skärsäter et al. (2005) [32] | Standard treatment in psychiatry | Not reported. | Patients received conventional psychopharmacological, counselling, and/or psychotherapeutic treatment immediately after the end of the investigation. | Not reported. | Therapists from psychiatry in Western Sweden. | Not reported. | Patients were followed up by their therapist during the first 6 months or longer if required. |
| Steig et al. (2023) [33] | Transdiagnostic group cognitive behavior therapy (UP-CBT) versus standard diagnosis specific  group cognitive behavior therapy (STD-CBT). | *Not reported in the paper, but in study protocol [34].*  Intervention group (UP-CBT): [35].  Standard group (STD-CBT): [36]. | Described as CBT. | Both groups received 14 weekly group therapy sessions. | Mental health professionals. | Groups. | Not reported. |
| Lawn et al. (2019) [37] | MindStep™ program | MindStep™, an innovative program in ‘Improving Access to Psychological Therapies’ (IAPT), a low intensity cognitive therapy intervention (LiCBT) associated with the creation of a new workforce to deliver early and low intensity interventions consistent with the National Institute for Health and Care Excellence (NICE) guideline recommendations. | Sessions followed a guided LiCBT format between coach and client, using the person’s own problem statements, goals, planned actions and workbook exercises, supported by guided self-help, and social prescribing to engage the person with their social networks and signposting to community services. A 6-month follow-up assessment was maintained to monitor improvements. | Delivered over 6-8 weeks. The average duration of an initial assessment was 1h, and duration of treatment sessions was approx. 30 min. | 7 LiCBT coaches delivered the program from Melbourne under close clinical supervision. Only one coach was a qualified mental health professional; others had health or community worker qualifications. | By telephone. | Clients were offered up to six treatment sessions. The MindStep^TM^ allowed flexible telephone delivery. |
| Woolley et al. (2020) [38] | Occupational therapy groups | The Recovery Workbook [39] was used in the LMI group, and the cognitive behavioural therapy intervention for individuals in supported employment [40] in the RTW group. | One group focused on facilitating return to work (RTW), and another group on strategies related to coping and living with mental illness (LMI). Group-specific criteria were motivation to make a change in their lives (LMI), and support needed to achieve a working or volunteering goal (RTW). | Both groups included weekly 2-hour sessions over 10 weeks. | Both groups were led by an occupational therapist. | Groups. | Participants who did not attend at least 75% of the group sessions were excluded. |
| Bouchal et al. (2023) [41] | Deep Brain Stimulation (DBS) and add-on cognitive behavior therapy (CBT) | CBT sessions were consistent with the Cognitive Therapy of Depression manual by Beck et al. [3]. | Patients with treatment-resistant depression received deep brain stimulation (DBS). Responders (≥50% reduction of HDRS-17 score from baseline) after 6-months of DBS, participated in CBT from 6 to 9 months and non-responders received CBT after the cross-over period from 12 to 15 months, irrespective of final responder status. Details are provided about the cross-over phase in the clinical trials paper [42]. | All participants received 12 add-on sessions of individual cognitive behavioral therapy during the trial. | CBT was performed by experienced cognitive behavioural therapists. | Individual. | Not reported. |

# **References**

1. Callesen, P., et al., *Metacognitive Therapy versus Cognitive Behaviour Therapy in Adults with Major Depression: A Parallel Single-Blind Randomised Trial.* Sci Rep, 2020. **10**(1): p. 7878.

2. Wells, A., *Metacognitive therapy for anxiety and depression*. 2011: Guilford press.

3. Beck, A.T., *Cognitive therapy and the emotional disorders*. 1979: Penguin.

4. Fennell, M.J.V., *Depression*, in *Cognitive behaviour therapy for psychiatric problems: A practical guide.* 1989, Oxford University Press: New York, NY, US. p. 169-234.

5. Craigie, M.A. and P. Nathan, *A nonrandomized effectiveness comparison of broad-spectrum group CBT to individual cbt for depressed outpatients in a community mental health setting.* Behavior Therapy, 2009. **40**(3): p. 302-314.

6. Barlow, D.H., M.G. Craske, and M.A. Mercier, *Mastery of your anxiety and panic II: client workbook*. 1999, Springer.

7. Nathan, P.R., et al., *Mood management course : a cognitive behavioural group treatment programme for anxiety disorders & depression*. 2001, Perth: Riobay Enterprises Pty. Ltd. (Publishing) Perth.

8. Ekeblad, A., et al., *Randomized Trial of Interpersonal Psychotherapy and Cognitive Behavioral Therapy for Major Depressive Disorder in a Community-Based Psychiatric Outpatient Clinic.* Depress Anxiety, 2016. **33**(12): p. 1090-1098.

9. Weissman MM, M.J., Klerman GL., *Comprehensive Guide To Interpersonal Psychotherapy*. 2000, New York, NY: Basic Books. 416 pages.

10. Christopher R. Martell, S.D., and Ruth Herman-Dunn, *Behavioral Activation for Depression: A Clinician’s Guide*. 2010, New York, NY: Guilford Press.

11. Tønning, M.L., et al., *The effect of smartphone-based monitoring and treatment on the rate and duration of psychiatric readmission in patients with unipolar depressive disorder: The RADMIS randomized controlled trial.* J Affect Disord, 2021. **282**: p. 354-363.

12. Thimm, J.C. and L. Antonsen, *Effectiveness of cognitive behavioral group therapy for depression in routine practice.* BMC Psychiatry, 2014. **14**: p. 292.

13. Roger Hagen, R.W.G., *Mestring av depresjon (manual)*. 2007, Trondheim, Norway: Tapir akademisk forl. 170.

14. Ezquiga, E., et al., *Factors associated with outcome in major depression: A 6-month prospective study.* Social Psychiatry and Psychiatric Epidemiology: The International Journal for Research in Social and Genetic Epidemiology and Mental Health Services, 1998. **33**(11): p. 552-557.

15. Ludman, E.J., et al., *Organized self-management support services for chronic depressive symptoms: A randomized controlled trial.* Psychiatric Services, 2016. **67**(1): p. 29-36.

16. Grote, N.K., et al., *Engaging women who are depressed and economically disadvantaged in mental health treatment.* Soc Work, 2007. **52**(4): p. 295-308.

17. Zuckoff, A., H.A. Swartz, and N.K. Grote, *Motivational interviewing as a prelude to psychotherapy of depression*, in *Motivational interviewing in the treatment of psychological problems.* 2008, The Guilford Press: New York, NY, US. p. 109-144.

18. Ludman, E.J., et al., *A pilot study of telephone care management and structured disease self-management groups for chronic depression.* Psychiatr Serv, 2007. **58**(8): p. 1065-72.

19. Cook, J.A., et al., *Results of a randomized controlled trial of mental illness self-management using Wellness Recovery Action Planning.* Schizophr Bull, 2012. **38**(4): p. 881-91.

20. Cook, J.A., et al., *A randomized controlled trial of effects of Wellness Recovery Action Planning on depression, anxiety, and recovery.* Psychiatric Services, 2012. **63**(6): p. 541-547.

21. Mueser, K.T., et al., *Illness management and recovery: a review of the research.* Psychiatr Serv, 2002. **53**(10): p. 1272-84.

22. Tutty, S., et al., *Evaluating the effectiveness of cognitive-behavioral teletherapy in depressed adults.* Behav Ther, 2010. **41**(2): p. 229-36.

23. Simon, G.E., et al., *Telephone psychotherapy and telephone care management for primary care patients starting antidepressant treatment: a randomized controlled trial.* Jama, 2004. **292**(8): p. 935-42.

24. Ludman, E.J., et al., *A randomized trial of telephone psychotherapy and pharmacotherapy for depression: continuation and durability of effects.* J Consult Clin Psychol, 2007. **75**(2): p. 257-66.

25. Bennett, G., *Miller, W. R. and Rollnick, S. (1991) Motivational interviewing: Preparing people to change addictive behavior. New York: Guilford Press, 1991. Pp. xvii + 348. £24.95 hardback, £11.50 paper. ISBN 0–89862–566–1.* Journal of Community & Applied Social Psychology, 1992. **2**(4): p. 299-300.

26. Rollnick, S. and W. Miller, *What is Motivational Interviewing?* Behavioural and Cognitive Psychotherapy, 1995. **23**: p. 325-334.

27. Vittengl, J.R., L.A. Clark, and R.B. Jarrett, *Moderators of continuation phase cognitive therapy's effects on relapse, recurrence, remission, and recovery from depression.* Behav Res Ther, 2010. **48**(6): p. 449-58.

28. *Adapting cognitive therapy for depression: Managing complexity and comorbidity*, in *Adapting cognitive therapy for depression: Managing complexity and comorbidity.*, M.A. Whisman, Editor. 2008, Guilford Press: New York, NY, US. p. xvi, 448-xvi, 448.

29. Jarrett, R.B., et al., *Preventing depressive relapse and recurrence in higher-risk cognitive therapy responders: a randomized trial of continuation phase cognitive therapy, fluoxetine, or matched pill placebo.* JAMA Psychiatry, 2013. **70**(11): p. 1152-60.

30. Fawcett, J., et al., *Clinical management--imipramine/placebo administration manual. NIMH Treatment of Depression Collaborative Research Program.* Psychopharmacol Bull, 1987. **23**(2): p. 309-24.

31. Vittengl, J.R., et al., *Longitudinal social-interpersonal functioning among higher-risk responders to acute-phase cognitive therapy for recurrent major depressive disorder.* J Affect Disord, 2016. **199**: p. 148-56.

32. Skärsäter, I., et al., *Sense of coherence and social support in relation to recovery in first-episode patients with major depression: A one-year prospective study.* International Journal of Mental Health Nursing, 2005. **14**(4): p. 258-264.

33. á Steig, D.H., et al., *Patient-reported outcome measures in depression.* Nordic Journal of Psychiatry, 2023. **77**(2): p. 212-219.

34. Arnfred, S.M., et al., *Transdiagnostic group CBT vs. standard group CBT for depression, social anxiety disorder and agoraphobia/panic disorder: Study protocol for a pragmatic, multicenter non-inferiority randomized controlled trial.* BMC Psychiatry, 2017. **17**(1): p. 37.

35. Bryde Christensen, A., et al., *Therapists' Perceptions of Individual Patient Characteristics that May Be Hindering to Group CBT for Anxiety and Depression.* Psychiatry, 2020. **83**(4): p. 344-357.

36. Barlow, D.H., L.B. Allen, and M.L. Choate, *Toward a unified treatment for emotional disorders.* Behavior Therapy, 2004. **35**(2): p. 205-230.

37. Lawn, S., et al., *Outcomes of telephone-delivered low-intensity cognitive behaviour therapy (LiCBT) to community dwelling Australians with a recent hospital admission due to depression or anxiety: Mindstep™.* BMC Psychiatry, 2019. **19**.

38. Woolley, H., et al., *"I'm not alone": Women's experiences of recovery oriented occupational therapy groups following depression.* Can J Occup Ther, 2020. **87**(1): p. 73-82.

39. Spaniol, L., Koehler, M., & Hutchinson, D., *The recovery workbook: Practical coping and empowerment strategies for people with psychiatric disability.* 1994, Boston University: Center for Psychiatric Rehabilitation, Sargent College of Health and Rehabilitation Sciences, Boston University.

40. Lecomte, T., M. Corbière, and P. Lysaker, *Une intervention de groupe cognitive comportementale pour les personnes suivies par un programme de soutien en emploi (TCC-SE).* L'Encéphale, 2014. **40**.

41. Raffin Bouchal, D.S., et al., *Personal recovery associated with deep brain stimulation for treatment-resistant depression: A constructivist grounded theory study.* J Psychiatr Ment Health Nurs, 2023. **30**(5): p. 1005-1018.

42. Ramasubbu, R., et al., *Long versus short pulse width subcallosal cingulate stimulation for treatment-resistant depression: a randomised, double-blind, crossover trial.* Lancet Psychiatry, 2020. **7**(1): p. 29-40.
