# Supplementary material for: Interventions promoting recovery from depression for patients transitioning from outpatient mental health services to primary care: A scoping review
Source: PLoS One. 2024 May 6;19(5):e0302229. doi: 10.1371/journal.pone.0302229 (PMC11073719; doi:10.1371/journal.pone.0302229)
Supplement: S9 Appendix — (DOCX) [file pone.0302229.s009.docx]

# **S9 Appendix**

## **Implementation strategies and modifications (*n* = 16)**

| Author and year | Implementation strategies | Modifications |
| --- | --- | --- |
| Callesen et al. (2020) [1] | Therapists received CBT supervision from a CBT therapist with 35 years of CBT experience. Supervision in MCT consisted of discussion of cases and observation of session video recordings. Before commencing the trial, each therapist treated a series of training cases under supervision and was provided by the originator of MCT. | Because one of the authors is the originator of MCT, they added an extra layer of quality assessment for CBT: a random sample of eight transcribed videotapes of CBT were sent to independent, leading practitioners in CBT and Fellows of the Academy of Cognitive Therapy for assessment of quality. |
| Craigie and Nathan (2009) [2] | Therapists received weekly routine clinical supervision provided by a senior clinical psychologist or the clinic director who had over 20 of years of CBT experience. | Individual CBT therapists implemented individual CBT in a more flexible manner based on a detailed cognitive behavioural care formulation developed for each patient. |
| Ekeblad et al. (2016) [3] | Therapists received training during 1–2 years in the psychotherapy method they used in the trial; IPT training 1–4 years and CBT training in 0–15 years prior to the trial. During the trial, therapists had regular supervision with certified supervisors, and had the opportunity to attend training seminars with teachers. Using videotapes from supervision was allowed, but not mandatory.  The IPT therapists were older (mean age 57.9 years) and had more years of professional experience in their basic profession compared to the CBT therapists were younger (mean age 37.6 years). | Not reported. |
| Tønning et al. (2021) [4] | Daily notifications reminded patients to fill out self-rating questions. In the case of several missing days, patients were contacted by a study nurse. Study nurses checked the patient self-rated data three times a week and contacted patients in case of several missing days, or reacted according to the data presented, providing a double feedback loop between the study nurse and the patient. | During the trial period, there were no long-lasting breakdowns. However, several times one or more functions were unavailable for a period of maximum 1-2 days. |
| Thimm and Antonsen (2014) [5] | Therapists received formal training in CBT. | As no single manual was used during the study period, there was some variation in the treatment received by groups. Group CBT sessions initially consisted of 12 weekly sessions but was later extended to 15 sessions. Each session lasted 120 minutes, including 15 minutes break. |
| Ezquiaga et al. (1998) [6] | Not reported | Medication could be changed to another antidepressant with a different pharmacologic profile if a response had not been obtained after 2 months. |
| Ludman et al. (2016) [7] | Intervention staff received weekly supervision by the study psychologist and psychiatrist. Care managers had experience working with patients with depression. Peer specialists completed a five-day training and certification program from the Depression and Bipolar Support Alliance. In addition, peer specialists referred clinical concerns to the care manager, treating providers, or supervisory team. Peer specialists held drop-in office hours and made group reminder calls which focused on goals and strategies to facilitate recovery. | Not reported. |
| Tutty et al. (2010) [8] | Not reported | Not reported. |
| Vittengl et al. (2010) [9] | Five therapists with a PhD in clinical psychology or MD (i.e., trained as a psychiatrist), completed ≥ 1 year of CT training and demonstrated competence (scores ≥ 40 on the Cognitive Therapy Scale (CTS); Young & Beck, 1980) before treatment. Therapists received weekly group supervision (and additional supervision when requested) during both acute and continuation-phase CT from a PhD clinical psychologist with extensive experience supervising CT. To facilitate treatment competence and adherence, an offsite consultant (a PhD clinical psychologist with extensive experience evaluating CT) reviewed videotapes of the 4^th^ and a randomly selected session of both acute and continuation-phase CT, scored therapists on the CTS, and provided them written feedback. All therapists achieved mean CTS scores > 40 during acute (grand M = 47.1, SE = 0.35) and continuation (grand M = 46.3, SE = 1.17) phase CT (Jarrett et al., 2001). Scores of 40 and above mark competence within both CT protocols. | Not reported. |
| Jarrett et al. (2013) [10] | Therapists completed at least one year of supervised CT training and demonstrated competence as documented by Cognitive Therapy Scale scores (CTS) of ≥ 40. Throughout the study, therapists received ongoing supervision or consultation; CTS ratings were made from randomly selected videotaped sessions. | During the first 6 years of the study, the primary goal was to test the efficacy of C-CT and FLX versus PBO across the 8-month experimental phase. Thereafter, the primary goal was to compare the durability of C-CT and FLX across 20 months post-randomization. |
| Vittengl et al. (2016) [11] | Cognitive therapists completed ≥1 year of CT training and demonstrated competence via CTS (Young & Beck, 1980) scores ≥ 40. Therapists submitted session videotapes for review and participated in weekly group supervision/feedback sessions. | During the first 6 years of the study, the primary goal was to test the efficacy of C-CT and FLX versus PBO across the 8-month experimental phase. Thereafter, the primary goal was to compare the durability of C-CT and FLX across 20 months post-randomization. |
| Skärsäter et al. (2005) [12] | Not reported | Not reported. |
| Steig et al. (2023) [13] | Therapists were certified and received CBT-training. | Not reported. |
| Lawn et al. (2019) [14] | Mental health coaches were trained and closely supervised by Flinders University mental health professionals who have postgraduate qualifications in CBT. Selected coaches received training that was conducted over 12-months using curriculum and competency standards (aligned with the UK IAPT services). | Not reported. |
| Woolley et al. (2020) [15] | Not reported | Not reported. |
| Bouchal et al. (2023) [16] | CBT was conducted by experienced cognitive behavioural  therapists. | Not reported. |

# **References**

1. Callesen, P., et al., *Metacognitive Therapy versus Cognitive Behaviour Therapy in Adults with Major Depression: A Parallel Single-Blind Randomised Trial.* Sci Rep, 2020. **10**(1): p. 7878.

2. Craigie, M.A. and P. Nathan, *A nonrandomized effectiveness comparison of broad-spectrum group CBT to individual cbt for depressed outpatients in a community mental health setting.* Behavior Therapy, 2009. **40**(3): p. 302-314.

3. Ekeblad, A., et al., *Randomized Trial of Interpersonal Psychotherapy and Cognitive Behavioral Therapy for Major Depressive Disorder in a Community-Based Psychiatric Outpatient Clinic.* Depress Anxiety, 2016. **33**(12): p. 1090-1098.

4. Tønning, M.L., et al., *The effect of smartphone-based monitoring and treatment on the rate and duration of psychiatric readmission in patients with unipolar depressive disorder: The RADMIS randomized controlled trial.* J Affect Disord, 2021. **282**: p. 354-363.

5. Thimm, J.C. and L. Antonsen, *Effectiveness of cognitive behavioral group therapy for depression in routine practice.* BMC Psychiatry, 2014. **14**: p. 292.

6. Ezquiga, E., et al., *Factors associated with outcome in major depression: A 6-month prospective study.* Social Psychiatry and Psychiatric Epidemiology: The International Journal for Research in Social and Genetic Epidemiology and Mental Health Services, 1998. **33**(11): p. 552-557.

7. Ludman, E.J., et al., *Organized self-management support services for chronic depressive symptoms: A randomized controlled trial.* Psychiatric Services, 2016. **67**(1): p. 29-36.

8. Tutty, S., et al., *Evaluating the effectiveness of cognitive-behavioral teletherapy in depressed adults.* Behav Ther, 2010. **41**(2): p. 229-36.

9. Vittengl, J.R., L.A. Clark, and R.B. Jarrett, *Moderators of continuation phase cognitive therapy's effects on relapse, recurrence, remission, and recovery from depression.* Behav Res Ther, 2010. **48**(6): p. 449-58.

10. Jarrett, R.B., et al., *Preventing depressive relapse and recurrence in higher-risk cognitive therapy responders: a randomized trial of continuation phase cognitive therapy, fluoxetine, or matched pill placebo.* JAMA Psychiatry, 2013. **70**(11): p. 1152-60.

11. Vittengl, J.R., et al., *Longitudinal social-interpersonal functioning among higher-risk responders to acute-phase cognitive therapy for recurrent major depressive disorder.* J Affect Disord, 2016. **199**: p. 148-56.

12. Skärsäter, I., et al., *Sense of coherence and social support in relation to recovery in first-episode patients with major depression: A one-year prospective study.* International Journal of Mental Health Nursing, 2005. **14**(4): p. 258-264.

13. á Steig, D.H., et al., *Patient-reported outcome measures in depression.* Nordic Journal of Psychiatry, 2023. **77**(2): p. 212-219.

14. Lawn, S., et al., *Outcomes of telephone-delivered low-intensity cognitive behaviour therapy (LiCBT) to community dwelling Australians with a recent hospital admission due to depression or anxiety: Mindstep™.* BMC Psychiatry, 2019. **19**.

15. Woolley, H., et al., *"I'm not alone": Women's experiences of recovery oriented occupational therapy groups following depression.* Can J Occup Ther, 2020. **87**(1): p. 73-82.

16. Raffin Bouchal, D.S., et al., *Personal recovery associated with deep brain stimulation for treatment-resistant depression: A constructivist grounded theory study.* J Psychiatr Ment Health Nurs, 2023. **30**(5): p. 1005-1018.
