# Supplementary material for: Interventions promoting recovery from depression for patients transitioning from outpatient mental health services to primary care: A scoping review
Source: PLoS One. 2024 May 6;19(5):e0302229. doi: 10.1371/journal.pone.0302229 (PMC11073719; doi:10.1371/journal.pone.0302229)
Supplement: Attachment — Submitted filename: Response to Reviewers.docx [file pone.0302229.s010.docx]

Dear Academic Editor Dr. Lakshit Jain

Thank you for your feedback and for providing us with the opportunity for revising our manuscript entitled "Interventions Promoting Recovery from Depression for Patients Transitioning from Hospital Psychiatry to Primary Care: A Scoping Review”. We thank the reviewers for their thorough evaluation of our manuscript. Please find a point-to-point reply to yours and the reviewers’ comments in the three tables below. The changes made to the manuscript are marked with track changes in the manuscript.

**Academic Editor**

| Comments from Editor | Answers from authors |
| --- | --- |
| Journal requirements:  When submitting your revision, we need you to address these additional requirements.  1. Please ensure that your manuscript meets PLOS ONE's style requirements, including those for file naming. The PLOS ONE style templates can be found at  [https://journals.plos.org/plosone/s/file?id=wjVg/PLOSOne_ formatting_sample_main_body.pdf](https://journals.plos.org/plosone/s/file?id=wjVg/PLOSOne_formatting_sample_main_body.pdf) and  [https://journals.plos.org/plosone/s/file?id=ba62/PLOSOne_ formatting_sample_title_authors_affiliations.pdf](https://journals.plos.org/plosone/s/file?id=ba62/PLOSOne_formatting_sample_title_authors_affiliations.pdf) | We have ensured that the manuscript meets PLOS ONE’s style requirements, including those for file naming. |
| 2. We suggest you thoroughly copyedit your manuscript for language usage, spelling, and grammar. If you do not know anyone who can help you do this, you may wish to consider employing a professional scientific editing service.  Whilst you may use any professional scientific editing service of your choice, PLOS has partnered with both American Journal Experts (AJE) and Editage to provide discounted services to PLOS authors. Both organizations have experience helping authors meet PLOS guidelines and can provide language editing, translation, manuscript formatting, and figure formatting to ensure your manuscript meets our submission guidelines. To take advantage of our partnership with AJE, visit the AJE website (<http://learn.aje.com/plos/>) for a 15% discount off AJE services. To take advantage of our partnership with Editage, visit the Editage website ([www.editage.com](http://www.editage.com)) and enter referral code PLOSEDIT for a 15% discount off Editage services.  If the PLOS editorial team finds any language issues in text that either AJE or Editage has edited, the service provider will re-edit the text for free.  Upon resubmission, please provide the following: The name of the colleague or the details of the professional service that edited your manuscript A copy of your manuscript showing your changes by either highlighting them or using track changes (uploaded as a *supporting information* file) A clean copy of the edited manuscript (uploaded as the new *manuscript* file)” | Thank you for your suggestions. The revised manuscript has been proofread by a colleague from out department, Siri Jonina Egede and her partner Luke James Norman. Luke James Norman is a native English speaker and Siri Jonina Egede has lived and worked in England for several years. |
| 3. We note that the grant information you provided in the ‘Funding Information’ and ‘Financial Disclosure’ sections do not match.  When you resubmit, please ensure that you provide the correct grant numbers for the awards you received for your study in the ‘Funding Information’ section. | We are sorry for the mistake. The correct grant information is listed in the ‘Funding Information’ section. |
| 4. When completing the data availability statement of the submission form, you indicated that you would make your data available on acceptance. We strongly recommend all authors decide on a data sharing plan before acceptance, as the process can be lengthy and hold up publication timelines. Please note that, though access restrictions are acceptable now, your entire data will need to be made freely accessible if your manuscript is accepted for publication. This policy applies to all data except where public deposition would breach compliance with the protocol approved by your research ethics board. If you are unable to adhere to our open data policy, please kindly revise your statement to explain your reasoning and we will seek the editor's input on an exemption. Please be assured that, once you have provided your new statement, the assessment of your exemption will not hold up the peer review process. | We are sorry for the mistake in the availability statement. Our raw data is available and was published at the Open Science Framework (OSF) database in connection with our submission of the article to PLOS ONE.  Please see line 645 in the manuscript:  All data generated or analysed during this scoping review is available online:  <https://osf.io/5894b/>  Line 161: A protocol was prepared in advance and registered in Open Science Framework (OSF) (<https://osf.io/ah3sv>), published in the medRxiv server (<https://doi.org/10.1101/2022.10.06.22280499>) and in PLOS ONE. |
| 5. We note that you have referenced (Young, J. and A.T. Beck, Cognitive therapy scale. Unpublished manuscript, University of Pennsylvania, 1980.) which has currently not yet been accepted for publication. Please remove this from your References and amend this to state in the body of your manuscript: (i.e., “Cognitive therapy scale.[Unpublished]”) as detailed online in our guide for authors [http://journals.plos.org/plosone/s/submission- guidelines#loc-reference-style](http://journals.plos.org/plosone/s/submission-guidelines#loc-reference-style) | Thank you very much for noticing this. The reference has been published. We have therefore inserted the correct reference in line 917 (reference number 109) in the manuscript. |
| 6. Please include a copy of Table 6 which you refer to in your text on page 23. | Thank you for noticing the missing table 6, p. 23. The manuscript does not include a table 6 but an appendix 9, which we have referred to later in the same section. Therefore, it is a mistake and we have deleted the reference to table 6. |
| 7. Please review your reference list to ensure that it is complete and correct. If you have cited papers that have been retracted, please include the rationale for doing so in the manuscript text or remove these references and replace them with relevant current references. Any changes to the reference list should be mentioned in the rebuttal letter that accompanies your revised manuscript. If you need to cite a retracted article, indicate the article’s retracted status in the References list and include a citation and full reference for the retraction notice. | Thank you for the reminder to revise the reference list. We have done that. |

**Reviewer #1**

| Comments from reviewer #1 | Answers from authors |
| --- | --- |
| Dear Dr. Lakshit Jain, I have had the honor of reviewing the article titled "Interventions Promoting Recovery from Depression for Patients Transitioning from Hospital Psychiatry to Primary Care: A Scoping Review” submitted to PLOS ONE for peer review. The manuscript addresses a very important topic, specifically an area of void where there are no studies extending the effectiveness of collaborative care approach in patients who received inpatient care for MDD and on discharge to ensure continuity of care and relapse prevention. To address this the authors took a scoping review approach. | Thank you very much for your positive response. |
| On line 84: The authors mentioned “To our knowledge, no studies have investigated collaborative care for patients who have been hospitalized with severe MDD” Recommendation: Please be specific that the authors are referring for patients with MDD transitioning into the community and the studies that lack are referring to the collaborative care on discharge for relapse prevention. | Thank you for your suggestion, we agree  that we could be more specific.  We have added a more specific text in the  manuscript in line 88:  *“To our knowledge, no studies have investigated collaborative care for relapse prevention for patients with MDD who have been discharged from outpatient mental health services (i.e., outpatient hospital-based mental health services, S1 Appendix) and continue their treatment in primary care”.* |
| On line 92: The authors claim the meaning of clinical recovery as “comprises full symptom remission” questionable if this is accurate as partial symptom improvement can also be considered as clinical recovery. | We agree that clinical recovery can also be considered and defined by authors as partial symptom improvement.  We have changed the text in the manuscript accordingly, in line 98:  *“Authors define clinical recovery as comprised partial or full symptom remission, independent living, gaining control over the illness, and full or part-time work or education”.* |
| Lines from 109-111: Please restructure the sentence as the existing terminology relays inconsistent and confusing message. | Thank you for noticing this. We agree that the sentence is unclear.  In the manuscript, in line 115-117, we have  added the following:  *“Research shows that most patients with MDD do not achieve symptomatic remission or full recovery after treatment in outpatient mental health services. Therefore, they are at risk of developing new episodes of MDD or treatment-resistant depression”.* |
| “Hospital psychiatry” term have been repeatedly used throughout the manuscript, recommend others to either change the wording or specify hospital psychiatric meaning inpatient psychiatric care. | Thank you for your comment. In our protocol article, we used the concept "mental health services". Therefore, we have decided to keep the concept from the protocol article. We have changed the concept in both the title of the article and throughout the manuscript from “hospital psychiatry” to “mental health services”. However, to make it clear that the transition of patients is from hospital-based outpatient psychiatry to primary care, we have added the concept and its precise definition to S1 Appendix: **Abbreviation/concept** \| **Definition**; Outpatient mental health services \| Outpatient hospital-based mental health services |
| Additional limitations as disclosed by the authors include the scoping review methodology itself where the focus is on the range of the literature versus the depth or validity of findings, the limitation of scoping review included inability to assisted equality or bias to provide a systematic assessment of the extreme validity of the evidence. As the others identified most of the literature is originated from Western countries which lacks diversity as it is not representing global population. | Thank you for sharing your reflection. We agree that it is a limitation. However, we have already written about these limitations in the discussion section (sub-section: “Strengths and limitations”).  Please see line 592-596 (limitations regarding scoping reviews as a method).  Please see line 601-604 (limitations regarding Western countries). In addition, we have added a final sentence to this theme in line 603: *“Thus, the review lacks diversity as it does not represent the global population”.* |

**Reviewer #2**

| Comments from reviewer #2 | Answers from authors |
| --- | --- |
| Thanks for giving me the opportunity to review this paper. The sample size of 1915 participants are great. I liked the paper but have some concerns. | Thank you very much for your positive response. |
| 1. Since there are no studies discussing interventions promoting recovery from depression for patients transitioning from hospital psychiatry to primary care is the title still relevant? | Thank you. We believe that the title is still relevant, as it expresses our aim and the research questions of the review and the perspectives, we investigate. Only few studies dealt with the very transitioning phase. However, we also included studies where patients were being prepared for transition at the end of their outpatient treatment being prepared for transition.  We have already mentioned this in the discussion section in the manuscript, line 532-536: *“However, we found limited knowledge about the key interest of this scoping review, i.e., studies about patients who had been treated in outpatient mental health services and were thereafter in a sector-transitioning phase. The studies investigated interventions either for patients in outpatient mental health services before discharge, or for patients after discharge, not specifically targeted to patients in the sector-transitioning phase”.* |
| 2. The use of different scales to measure recovery in depression makes it difficult to define recovery in the population that was studied. | Thank you for your reflection. We agree that that the use of different recovery scales makes it difficult to measure and define recovery. It was one of our findings that there was a broad variation in scales and definitions. We recommend future research to investigate this further. In the section “Implications for research” (p. 32 in the manuscript) we have added in line 614:  *“In addition, we identified a variety of scales to measure recovery, which makes it difficult to measure and define recovery in patients with MDD. Therefore, we recommend that future recovery research will investigate this further”.* |
| 3. It would be interesting to know which antidepressant medications recovered patients were on. | We agree that it would be interesting to know which anti-depressant medication the recovered patients received. We did not mention the names of the medication in the review, but they can be found in the uploaded material in our online raw-data extraction file. |
| 4. The intervention e.g., CBT varied in terms of methods of delivery and length of delivery. It would be great to see if there is a specific length of time CBT should be delivered that would be conducive to recovery i.e., coming up with standard guidelines and length of treatment. | Thank you for your comment. Information on methods of delivery and length of delivery is included in S8 Appendix. However, the influence of different forms of delivery cannot be examined in a scoping review. |
| 5. Psychodynamic psychotherapy is a useful modality to explore underlying trauma, conflicts and childhood adverse experiences that drive depression. It would be great to see if it can be used in conjunction with CBT to facilitate long term recovery and address the causes of depression which may be deep rooted in childhood experiences, trauma, and unconscious conflicts. It helps by making the patients aware of these unconscious conflicts and promoting lasting change. | Thank you. We have added the following on line 549-552 in the manuscript:  *“No studies used psychodynamic psychotherapy. Those studies that used psychotherapy all used elements of CBT. Overall, the interventions were predominantly delivered to groups or by telephone”.* |
| Minor mistakes: Row 416 "Versus" should be removed at the end or maybe it is an incomplete sentence, and something needs to be added Row 971, 972: the reference 143 is in a language other than English | Thank you for noticing these mistakes.  We have removed the “versus” at the end of the sentence (line 416).  Regarding reference 143 (line 998): The Abstract is both in France and in English ([https://www.sciencedirect.com/science/article /abs/pii/S0013700614001109](https://www.sciencedirect.com/science/article/abs/pii/S0013700614001109) ).  The reference is mentioned in S8 Appendix: Content of interventions. The reference is included in one of our included studies from the literature search (Wooley et al. 2020). Wooley et al. (2020) describe that their material used in their intervention was inspired by:  *“The Recovery Workbook (143) was used in the LMI group, and the cognitive behavioural therapy intervention for individuals in supported employment (144) in the RTW group”.*  Please see S8 Appendix. |

**Reviewer #3**

| Comments from reviewer #3 | Answers from authors |
| --- | --- |
| **Strengths of the manuscript:**  -The abstract clearly describes the findings of the study. -The introduction to the article is well laid out. -Methods and results are clearly explained. The tables included in the manuscript help to review the studies quickly. -The discussion and conclusion further elaborate on the study findings and suggestions for future research. | Thank you very much for your positive evaluation of the strengths of the manuscript. |
| **Some recommendations are:** -After reading the manuscript title, “Hospital psychiatry,” my initial impression was that the authors are reviewing studies of patients diagnosed with depression transitioning from in-patient settings (e.g., psychiatric hospitals or general hospitals/medical floor, etc.) to outpatient primary care providers. However, the authors use “Hospital psychiatry”, in the title and throughout the manuscript, interchangeably when referring to outpatient and/or in-patient psychiatric care settings, which seems inaccurate. In the study protocol published in 2023, the authors have used the term “outpatient mental health services” in the title. I would appreciate it if the authors could add brief commentary to clarify. | Thank for your comment. We agree, and we have changed the term from “hospital psychiatry” to “outpatient mental health services”, corresponding to the term that we used in our scoping review protocol article. Please see response to Reviewer #1. |
| -The authors have primarily included efficacy/effectiveness trials, discussed intervention outcomes, and some studies on variables that may influence intervention outcomes. There are multiple systematic, meta-, and network analyses published comparing different interventions for depression. I will recommend if the authors could add brief commentary discussing how their manuscript is unique compared to previously published studies. The assertion that this is the “first" manuscript to report such findings seems misleading and inaccurate. | Thank you for your statement. We aimed to conduct a scoping review, i.e., to provide a descriptive overview of the existing evidence on interventions that promote recovery from MDD for patients transitioning from outpatient mental health services to primary care. We included studies where patients were in the transitioning phase or at the end of their outpatient treatment being prepared for transition. We did not include systematic reviews or meta-analysis of studies comparing different treatments of depression. However, we conducted a systematic search. In our PRISMA-flow chart, fig. 1 on page 12 in the manuscript, we describe reasons for exclusion of articles based on full-text screening. The primary reasons for exclusion were wrong setting, e.g., the intervention was conducted exclusively in primary care without a preceding treatment in hospital psychiatry, followed by wrong outcome, i.e., articles did not include “recovery” as an outcome measure. In addition, most articles collected data from different populations, e.g., both unipolar depression, bipolar depression, and schizophrenia. In our PICO we defined that we only included studies with populations diagnosed with unipolar depressive disorder. |
| -Line 481: typo “...expect for the DBS” | Thank you for noticing the typo. We have corrected this and added it on line 504:  *“…except for the DBS study”.* |
